# Finding the keys to successful adult-targeted advertisements on obesity prevention: an experimental audience testing study

**DOI:** 10.1186/s12889-015-2159-6

**Published:** 2015-08-20

**Authors:** Helen Dixon, Maree Scully, Sarah Durkin, Emily Brennan, Trish Cotter, Sarah Maloney, Blythe J. O’Hara, Melanie Wakefield

**Affiliations:** Centre for Behavioural Research in Cancer, Cancer Council Victoria, 615 St Kilda Road, Melbourne, Victoria 3004 Australia; World Lung Foundation, 61 Broadway, Suite 2800, New York, NY 10006 USA; Prevention Research Collaboration, Sydney School of Public Health, The University of Sydney, Level 2, Medical Foundation Building, K25, The University of Sydney, Sydney, New South Wales 2006 Australia

## Abstract

**Background:**

Mass media communications are an important component of comprehensive interventions to address population levels of overweight and obesity, yet we have little understanding of the effective characteristics of specific advertisements (ads) on this topic. This study aimed to quantitatively test audience reactions to existing adult-focused public health television ads addressing overweight and obesity to determine which ads have the highest levels of message acceptance, argument strength, personalised perceived effectiveness and negative emotional impact.

**Methods:**

1116 Australian adults aged 21-55 years recruited from a national online panel participated in this web-based study. Quotas were applied to achieve even numbers of males and females, those aged 21-29 years and 30-55 years, and those with a healthy weight (BMI = 18.5-24.9) and overweight/obesity (BMI = 25+). Participants were randomly assigned to view and rate four of eight ads that varied in terms of message content (health consequences, supportive/encouraging or social norms/acceptability) and execution style (graphic, simulation/animation, positive or negative testimonial, or depicted scene).

**Results:**

*Toxic fat* (a graphic, health consequences ad) was the top performing ad on all four outcome measures and was significantly more likely than the other ads tested to promote strong responses in terms of message acceptance, argument strength and negative emotional impact. *Measure up* (a negative testimonial, health consequences ad) performed comparably on personalised perceived effectiveness. Most ads produced stronger perceptions of personalised perceived effectiveness among participants with overweight/obesity compared to participants with healthy weight. Some ads were more likely to promote strong negative emotions among participants with overweight/obesity.

**Conclusions:**

Findings provide preliminary evidence of the most promising content and executional styles of ads that could be pursued as part of obesity prevention campaigns. Ads emphasising the negative health consequences of excess weight appear to elicit stronger cognitive and emotional responses from adults with overweight/obesity. However, careful pre-testing of these types of ads is needed prior to their inclusion in actual campaigns to ensure they do not have unintended negative impacts such as increased stigmatisation of vulnerable individuals and increased levels of body dissatisfaction and/or eating-disordered behaviour among at-risk population sub-groups.

**Electronic supplementary material:**

The online version of this article (doi:10.1186/s12889-015-2159-6) contains supplementary material, which is available to authorized users.

## Background

With the increasing prevalence of obesity globally [1], there is an urgent need for public health interventions to reduce and prevent obesity and its associated health problems. Since obesity is caused by a combination of individual, environmental, biological and genetic factors, no single intervention can reasonably be expected to have a substantial impact on obesity rates. However, in concert with other strategies, mass media campaigns offer a promising means of reaching target populations with obesity prevention messages [2]. A recent review of public health mass media campaigns highlighted their capacity to produce positive changes or prevent negative changes in health-related behaviours across large populations [3]. As excess body weight occurs in part due to an energy imbalance, influenced by modifiable lifestyle behaviours of increased activity and reduced dietary intake [4], encouraging and supporting these behaviours is a necessary component of achieving population level weight change.

While there has been a long history of mass media campaigns in areas such as tobacco control [5, 6] and skin cancer prevention [7, 8], the use of such campaigns to address overweight and obesity is a newer phenomenon. In Australia, the first national obesity mass media campaign, *Measure up*, was launched in 2008 [9], with a smaller state-based campaign highlighting the relationship between obesity and cancer (*Piece of string*) running in the preceding year [10]. Internationally, there are a few earlier examples of campaigns specifically targeting obesity such as *Fighting fat, fighting fit* which was initiated in the UK in 1999, and *Maat je niet dik!* [translated literally means “Don’t get fat!”] which ran in the Netherlands from 2002. Since 2009, there has been a noticeable shift towards greater investment by governments and public health organisations in obesity prevention mass media campaigns (e.g. *Pouring on the pounds* and *Strong4Life* in the United States, *Change4Life* in the UK, and *Swap it, don’t stop it* and *LiveLighter* in Australia). Given finite resources, it is crucial that funds are channelled into campaigns either known to be effective in promoting awareness or behaviour change or with the greatest potential for effectiveness.

Previously conducted research to inform or evaluate individual obesity prevention campaigns provides useful data to help refine education messages and understand whether campaign objectives have been achieved. However, it is challenging to assess the relative impact of these campaigns and identify the advertising messages and executional styles that appear most influential due to variations in media buys, audiences and evaluation measures. Advertisement (ad) rating studies conducted with smoking cessation ads have provided important insights into the types of messages that are likely to be most effective with smokers [11–15]. Drawing on this methodological approach, the present study aimed to build new knowledge by directly comparing existing television ads addressing obesity prevention using equal levels of exposure and a standard protocol with quantitative measures. A secondary aim of the study was to examine whether audience responses to each ad differed as a function of personal weight status.

## Method

### Design and participants

A web-based method was employed whereby adults were randomly allocated to view and rate four of eight obesity prevention ads. Participants were Australian residents recruited from a national online panel, comprising members initially sourced in a variety of ways such as computer-assisted telephone interviews, face-to-face research and online market research databases. Panel members were sent an email with a web link to the survey, inviting them to participate in the study. Upon accessing the survey website, a series of screening questions confirmed that participants met the following eligibility criteria: aged 21-55 years; not underweight (body mass index (BMI) of 18.5 or higher based on self-reported height and weight); and not employed (or have close family or friends) in advertising, or as dieticians, nutritionists or fitness instructors. These occupation groups were excluded as expertise in the topic area could potentially bias responses to the ads. Quotas were applied to achieve even numbers in key audience sub-groups of interest (i.e. males and females; those aged 21-29 years (young adults) and 30-55 years (middle-aged adults); those with healthy weight (BMI: 18.5-24.9) and overweight/obesity (BMI: 25.0 or higher) persons). Based on results from previous experiments testing smoker’s responses to anti-smoking ads [11, 13], we aimed to detect a difference of 10 % in the proportion of participants that perceived different ads to be acceptable or effective (e.g. 55 % of those exposed to ad A versus 45 % of those exposed to ad B). Power calculations showed that a sample size of 1000 (i.e. 500 ratings per ad) would allow the detection of such a difference in proportions with power of 0.87 (P = 0.05). Ethical approval to conduct the study was obtained from Cancer Council Victoria’s Institutional Research Review Committee. Implied consent was obtained by panel members clicking on the web link and completing the survey.

### Advertisements

A total of eight ads were selected from a sample of 99 ads catalogued as part of a scoping study of recent English-language obesity prevention and lifestyle television ads [16]. Relevant ads that had previously aired on television were identified through internet search engine Google and video sharing sites such as YouTube, as well as via the websites of government agencies and health organisations. Each ad was initially coded for message content and execution style. The three categories of message content were: health consequences (i.e. providing health arguments/evidence on *why* people should change their behaviour); supportive/encouraging (i.e. advising *how* to make and/or encouraging positive behaviour changes); or social norms/acceptability (i.e. promoting notion that healthy behaviours are socially desirable or acceptable, or that unhealthy behaviours are socially undesirable). Execution style was classified as follows: graphic (e.g. pictures of diseased organ); simulation/animation (e.g. animated characters, models of people or foods); positive testimonial (e.g. personalised narrative of how they lost weight through healthy eating and exercise); negative testimonial (e.g. personalised narrative of how they became obese and developed health problems); or depicted scene (e.g. actors portraying a scenario or series of events). Four members of the research team, all with health communication expertise, then viewed each identified ad and selected eight that (i) focused on obesity prevention and (ii) were deemed culturally relevant or have potential to be adapted for Australian audiences (e.g. changing end-frame, using Australian voice-over). Through this process, the primary consideration was to choose ads that were judged as likely to be most effective from each of the three message content types. A secondary consideration was to ensure that a mix of execution styles was achieved. Table 1 outlines the message content and executional style of each of the eight ads tested (note that the titles of the ads were not provided to participants). See Additional file 1 for detailed ad descriptions.

**Table 1:** Characteristics of obesity prevention advertisements tested

Ad name	Country/Region	Length	Message content	Execution style
Become a swapper	Australia	45 s	Supportive/encouraging	Simulation/animation
Toxic fat	Australia	30 s	Health consequences	Graphic
Take life on	Scotland	30 s	Supportive/encouraging	Positive testimonial
Measure up	Australia	60 s	Health consequences	Negative testimonial
Piece of string	Australia	30 s	Health consequences	Depicted scene
Full monty	Scotland	30 s	Social norms/acceptability	Depicted scene
Correctly identified	USA	30 s	Social norms/acceptability	Depicted scene
Why am I fat	USA	30 s	Social norms/acceptability	Depicted scene

### Procedure and measures

Questions assessing qualifying criteria and quotas were completed initially. Those eligible for the study went on to view and rate four ads and complete additional demographic questions (level of educational attainment and parental status). The order in which participants viewed their four ads was randomised to avoid sequence effects. Eligible participants were shown their first randomly assigned ad twice, and then asked to complete a series of ad rating questions. This process was repeated for the remaining three ads. Before any ads were shown, participants were instructed that some of the ads may be from different countries and that when watching the ads they should focus on their main messages and imagery, rather than cultural differences such as accents. The ad rating questions were adapted from other studies which have shown them to be sensitive indicators of advertising effectiveness [11, 13, 14, 17–19].

Cognitive responses were measured by asking participants to indicate the extent to which the ad ‘was believable’, ‘was relevant to me’, ‘made me stop and think’, ‘taught me something new’, ‘was easy to understand’, ‘was effective’, ‘made a strong argument for being a healthy weight’, ‘made me feel concerned about my weight’, ‘made me motivated to take action to achieve or stay a healthy weight’ and ‘made me feel confident to work on reaching or staying a healthy weight’. Responses to these items were recorded on five-point Likert scales where 1 = ‘strongly disagree’, 2 = ‘somewhat disagree’, 3 = ‘neither agree nor disagree’, 4 = ‘somewhat agree’ and 5 = ‘strongly agree’. Negative emotional reactions were measured by asking participants to indicate the extent to which watching the ad made them feel ‘disgusted’, ‘anxious’, ‘ashamed’, ‘fearful’, ‘guilty’ and ‘sad’, with responses recorded on a seven-point Likert scale ranging from 1 = ‘not at all’ to 7 = ‘extremely’. To avoid order effects, items within each section were presented randomly.

After rating each ad individually, participants were presented with screenshots of their four ads simultaneously on screen and asked to indicate which one of these ads they would be MOST likely to mention to someone else (since interpersonal communication surrounding health campaigns can stimulate change through the communication of social norms and social diffusion) [20], and which ad would be MOST likely to motivate them to change their lifestyle.

### Statistical analysis

Data were analysed using Stata/SE 12.1 (StataCorp, Texas, USA). Preliminary principal component analyses were conducted, averaging across individuals’ ratings for their four assigned ads, to explore whether the ad rating items could be reduced to a parsimonious set of outcomes. Based on the results of these exploratory analyses, as well as consideration of ad rating outcomes used in past research, four composite scales were computed. The first scale, labelled message acceptance (α = 0.889) was made up of two items: understandable and believable. The second scale, called argument strength (α = 0.883), also included two items: strong argument and effective. The third scale, labelled personalised perceived effectiveness (α = 0.915), comprised six items: relevant; stop and think; taught something new; concerned; motivated to take action; and confident to work on. The fourth scale, referred to as negative emotional impact (α = 0.950), included all six negative emotional reactions items. For analysis purposes, scores on the four scales were dichotomised at > 3.5 for message acceptance, argument strength and personalised perceived effectiveness, and > 4.5 for negative emotional impact to indicate strong responses.

To explore whether emotional responses related to cognitive responses, chi-square tests between negative emotional impact and message acceptance, argument strength and personalised perceived effectiveness respectively, were performed. These tests were run using the whole sample and then separately for the sub-group with overweight/obesity, with phi coefficients calculated to determine the strength of association.

Multivariate logistic regression analyses, using robust errors to account for the same individual rating multiple ads, were performed to compare the eight ads on each outcome. The average predicted probabilities of strong responses for all ads on all outcomes were calculated over all observations. Significance testing was conducted of the highest and then lowest rated ad on each outcome compared with all other ads. Further multivariate logistic regression analyses were conducted on the overall sample of participants, in order to examine any differences in participants’ ad ratings by weight status (healthy weight cf. overweight/obese) for the four outcomes, separately for each of the eight ads. All multivariate analyses controlled for gender, age (21-29 years or 30-55 years), parental status (parent/carer of child aged under 18 who lives with you or not), education level (completed tertiary education (i.e. university, technical and further education, or college) or not), weight status and whether or not participants had previously seen the ad before completing the online survey. Analyses comparing ratings of each ad by weight status used a more stringent level of statistical significance (P < 0.01) to account for multiple testing.

The proportion of participants who nominated a given ad (from their set of four ads) as the one they were MOST likely to: ‘mention to someone else’; and ‘motivate them to change their lifestyle’ were also calculated, with divergence above or below 12.5 % (i.e. chance) examined.

## Results

### Sample characteristics

A total of 1116 participants completed the online survey, of which 49 % were classified as having healthy weight (n = 542) and 51 % having overweight or obesity (n = 574). Overall, there was a relatively even split of males and females in our sample (50 % each), reflecting the population gender distribution for persons aged 21-55 years [21]. The proportion of 21-29 year olds in our sample (49 %) was considerably higher than the population (27 %) [21], due to the use of age quotas during study recruitment. Nearly half (49 %) of all participants had completed tertiary education, while 42 % indicated they were a parent/carer of a child under 18 years. Participants with overweight/obesity were more likely to be parent/carers compared to participants of healthy weight (45 % cf. 38 %), and less likely to be tertiary-educated participants (44 % cf. 54 %).

### Associations between ad rating outcomes

Across the whole sample, there were weak positive correlations between negative emotional impact and message acceptance (Phi = 0.08; P < 0.001) and argument strength (Phi = 0.14; P < 0.001) respectively, and a slightly stronger positive relationship between negative emotional impact and personalised perceived effectiveness (Phi = 0.25; P < 0.001). Similar effect sizes were found for the sub-group with overweight/obesity (message acceptance: Phi = 0.12; argument strength: Phi = 0.19; personalised perceived effectiveness: Phi = 0.25; all P’s < 0.001).

### Ad rating outcomes

As shown in Table 2, *Toxic fat* generated strong responses among the highest proportion of respondents on all four outcome measures. This ad was significantly more likely than all other ads to produce a strong response on message acceptance, argument strength and negative emotional impact. *Measure up* was the only ad to perform comparably (i.e. not significantly different) to *Toxic fat* with respect to personalised perceived effectiveness. *Correctly identified* was the lowest rated ad for two of the four outcome measures (message acceptance and argument strength), and was not significantly different from the lowest rated ad on negative emotional impact (*Take life on*). *Why am I fat* was the lowest rated ad for personalised perceived effectiveness, closely followed by *Full monty*.

**Table 2:** Average predicted probabilities (95 % confidence intervals) of strong ad responses

	Message acceptance		Argument strength		Personalised perceived effectiveness		Negative emotional impact	
	%	(95 % CI)	%	(95 % CI)	%	(95 % CI)	%	(95 % CI)
Become a swapper	*70*	*(65-74)*	54	(50-59)	40	(36-45)	*5*	*(3-7)*
Toxic fat	**83** ^**a**^	**(80-86)**	**76** ^**a**^	**(72-79)**	**51** ^**a**^	**(47-55)**	**27** ^**a**^	**(23-31)**
Take life on	73	(70-77)	57	(53-61)	40	(36-44)	*4^*	*(3-6)*
Measure up	78	(74-82)	69	(64-73)	**50**	**(46-55)**	17	(13-21)
Piece of string	78	(74-81)	69	(65-72)	45	(41-49)	12	(10-15)
Full monty	74	(70-77)	56	(52-60)	*27*	*(24-31)*	10	(8-13)
Correctly identified	*65^*	*(61-69)*	*36^*	*(32-40)*	29	(25-33)	*6*	*(4-8)*
Why am I fat	*69*	*(65-72)*	53	(49-57)	*23^*	*(20-27)*	14	(11-17)

Footnote: Percentages are adjusted for gender, age group, education level, parental status, weight status, previous exposure to the ad, all other ads and individual-level clustering. For each outcome (column) the ad that produced a strong response among the highest proportion of participants is indicated by ^a^ and **bold**; the ad that produced a strong response among the lowest proportion of participants is indicated by ^ and *italic*. **Bold** figures highlight those ads that were rated comparably to the strongest performing ad for each outcome (non-significant difference, P ≥ 0.05). *Italic* figures highlight those ads that were rated comparably to the weakest performing ad for each outcome (non-significant difference, P ≥ 0.05)

### Comparison of ad ratings by weight status

Participants with overweight/obesity were consistently more likely to experience a strong personalised perceived effectiveness response than participants of healthy weight, with the exception of *Correctly identified* (see Table 3). Those with overweight/obesity were also more likely to experience strong negative emotions in response to *Toxic fat*, *Measure up* and *Piece of string*. All ads were rated comparably by participants with healthy weight and overweight/obesity for message acceptance and argument strength.

**Table 3:** Adjusted odds ratios (95 % confidence intervals) for associations between weight status and strong ad responses

	Message acceptance		Argument strength		Personalised perceived effectiveness		Negative emotional impact	
Become a swapper	0.92	(0.61-1.38)	0.92	(0.65-1.32)	2.62^**^	(1.82-3.75)	1.20	(0.55-2.63)
Toxic fat	1.11	(0.71-1.74)	1.24	(0.84-1.85)	2.16^**^	(1.52-3.06)	2.42^**^	(1.62-3.61)
Take life on	1.00	(0.68-1.46)	1.13	(0.80-1.59)	2.30^**^	(1.60-3.30)	1.56	(0.64-3.85)
Measure up	1.49	(0.95-2.35)	1.48	(1.00-2.18)	3.23^**^	(2.24-4.65)	2.27^*^	(1.39-3.72)
Piece of string	0.90	(0.60-1.35)	1.07	(0.74-1.54)	2.01^**^	(1.42-2.85)	2.33^*^	(1.35-4.01)
Full monty	0.97	(0.67-1.42)	1.03	(0.73-1.44)	2.33^**^	(1.57-3.45)	1.51	(0.86-2.63)
Correctly identified	1.05	(0.74-1.49)	0.85	(0.59-1.21)	1.57	(1.07-2.31)	1.53	(0.73-3.20)
Why am I fat	1.20	(0.83-1.72)	0.86	(0.61-1.22)	1.74^*^	(1.15-2.63)	1.29	(0.80-2.09)

Footnote: For all models, healthy weight is the reference category (1.00). Significant difference between groups *P < 0.01; **P < 0.001. Analyses adjusted for gender, age group, education level, parental status, and previous exposure to the ad

### Overall ad choice

**Fig. 1:**
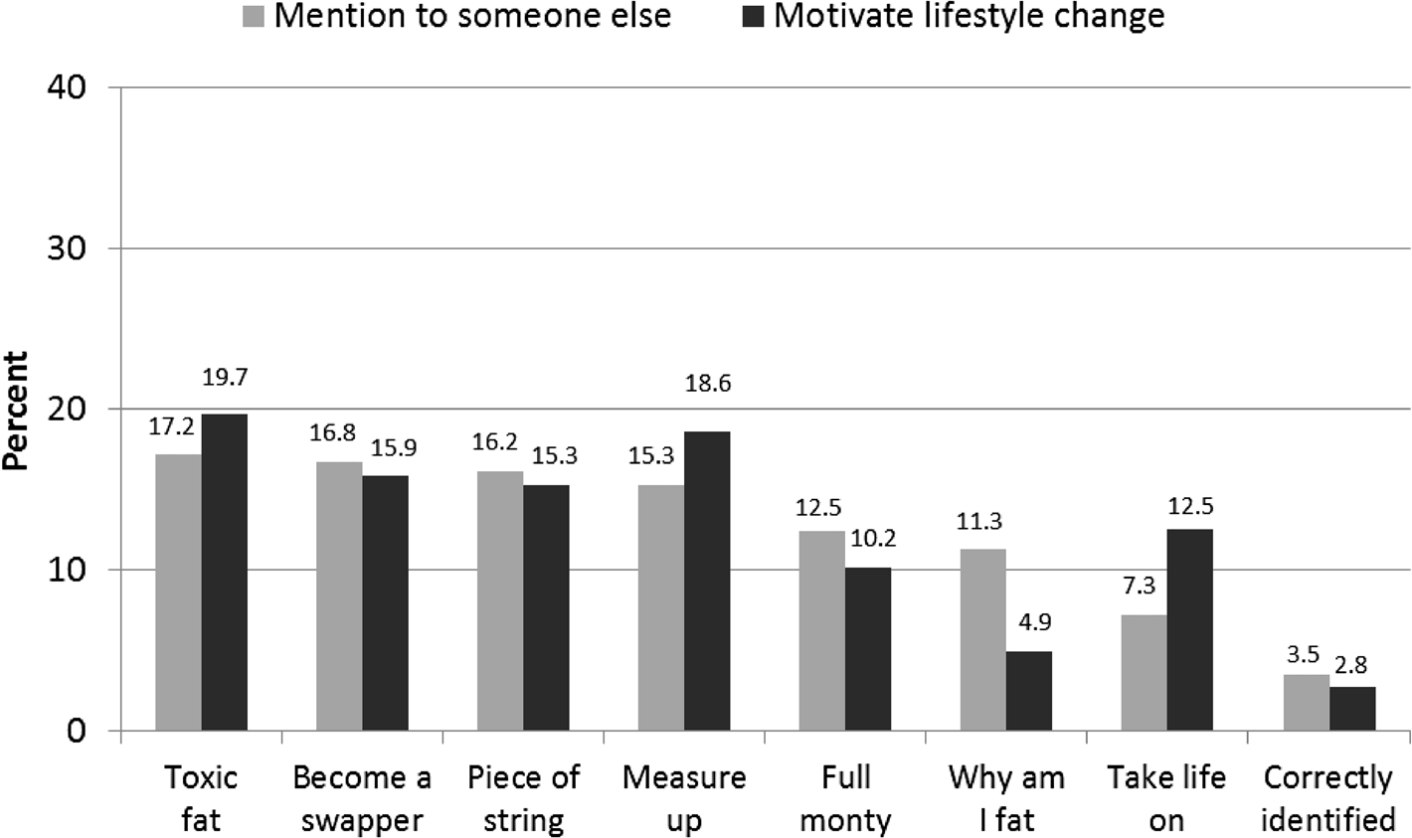
Frequency of ads selected by participants as most likely to mention to someone else and motivate them to change their lifestyle

As Fig. 1 indicates, all of the health consequences ads (*Toxic fat*, *Piece of string*, *Measure up*) along with *Become a swapper* were selected more frequently than by chance by participants both in terms of being most likely to be mentioned to someone else and to motivate lifestyle change. Participants tended to identify *Why am I fat* as an ad that would most likely prompt discussion rather than a shift in behaviour whereas the opposite pattern was observed for *Take life on*. Few participants (lower than chance) selected *Correctly identified* as the ad they would most likely mention to someone else or that would be most likely to motivate them to change their lifestyle.

## Discussion

This study aimed to provide evidence on the types of television ad messages and execution styles that provide most promise to pursue as part of mass media campaigns directly addressing obesity prevention. Of the eight ads tested, *Toxic fat* was most likely to produce strong responses on the four primary outcome measures, with only *Measure up* performing equivalently to it in terms of personalised perceived effectiveness. Both of these ads communicated health consequences messages – the first via a graphic execution, and the second via a negative personal testimonial. In general, ads containing messages regarding social norms/acceptability (*Why am I fat*, *Full Monty* and *Correctly identified*) were least likely to elicit a strong response for personalised perceived effectiveness while, as would be expected, supportive/encouraging ads (*Become a swapper* and *Take life on*) tended to be less likely to elicit a strong negative emotional impact. The top ranking ad (*Toxic fat*), performed comparatively more strongly on each outcome compared to each of the ads featuring depicted scenes (*Piece of string*, *Full Monty*, *Correctly identified* and *Why am I fat*). Notably, all but one of the ads (*Correctly identified*) produced stronger perceptions of personalised perceived effectiveness among participants with overweight/obesity compared with participants with healthy weight. The latter finding is encouraging as it suggests the ads promoted empowering responses, including feeling motivated and confident to take action, among participants with overweight/obesity.

To our knowledge, this is the first published study to systematically test the relative performance of existing television ads that directly addressed obesity prevention, rather than focusing on lifestyle behaviours (diet and activity) in isolation. The finding that the top two rated ads (*Toxic fat* and *Measure up*) both contained messages about health consequences is consistent with tobacco control advertising research indicating that smoking cessation ads with negative health effects messages, which typically use testimonials or graphic executions, are most effective at generating increased knowledge, beliefs, perceived effectiveness, and quitting behaviour [5]. However, it is possible that other unmeasured elements of these two ads such as the use of graphic imagery in *Toxic fat* and a personal narrative in *Measure up* may, at least in part, be contributing to their comparatively higher ratings. Further audience testing research with a larger sample of ads for each message type and executional style is needed before any strong conclusions can be made about the relative superiority of obesity prevention ads that emphasise the health consequences of excess weight compared to those that provide support and/or encouragement for lifestyle behaviour changes or focus on social norms/acceptability.

On the whole, ads tested in our study generated relatively low levels of emotional engagement overall, albeit with some variability. The ad that induced a strong negative emotional response from the highest proportion of participants was also the best performing ad on the remaining three outcomes. We also found evidence of a weak-moderate positive relationship between negative emotional impact and personalised perceived effectiveness, suggesting that generating some level of discomfort may be tied with perceptions of ad effectiveness. This hypothesis is further supported by research that demonstrates that anti-smoking ads with strong emotion activation or graphic images are most effective at prompting quitting behaviour [22–24]. Longitudinal research is needed to examine whether obesity prevention ads that consumers report to be persuasive but that also evoke negative emotions actually promote positive and sustained lifestyle and nutrition behaviour changes in the broad population. While promoting some level of concern about excess body weight may motivate healthy eating and activity levels, it is important to ensure that such messages do not inadvertently promote excessive body dissatisfaction which could result in disordered eating or unhealthy dieting practices in some individuals (see [25, 26]).

It was encouraging to note that the majority of ads tested were more likely to be rated strongly for personalised perceived effectiveness by people with overweight or obesity. Perceived message effectiveness has been shown to be causally related to attitude and intention change making it a useful indicator of the persuasiveness of ads [14, 17, 19, 27]. From audience research with anti-smoking ads, there is also evidence that measures of perceived effectiveness that specifically tap the extent to which a smoker is personally affected by the message (i.e. personalised perceived effectiveness) predict subsequent changes in quitting intentions and smoking behaviour [18]. It would be beneficial for future studies to establish a similar association between ratings of personalised perceived effectiveness for obesity prevention ads and behaviour change following exposure, in order to validate the utility of this rating measure when testing obesity prevention ads.

### Ethical considerations

While our study indicates there may be value in developing obesity prevention ads that elicit negative emotions, this finding needs to be balanced against ethical concerns raised by a number of public health scholars regarding the possible adverse effects of these types of messages [28–30]. For example, ads that frame overweight as an individual issue may inadvertently place blame on people with overweight and impact their emotional and physical well-being [31, 32]. Previous research points to a tendency for individuals who feel ashamed or stigmatised about their weight to engage in unhealthy eating patterns (e.g. binge eating) that can reinforce weight gain and impair weight loss [32]. A US study assessing adult reactions to obesity-related campaign slogans found that those that focused on diet and/or physical activity without reference to body weight received the most favourable responses and were rated as most likely to motivate health behaviour change, while messages seen as stigmatising individuals with overweight were rated less favourably and as less motivating [33]. Similarly, results from a randomised controlled trial in the US that tested reactions to print campaign materials indicated that campaigns that had been pre-tested and publicly criticised as stigmatising of people with obesity induced less self-efficacy than campaigns with more neutral content [34]. An important limitation of our study was the absence of a measure of the degree to which participants perceived the ads to be stigmatising and the degree to which they were associated with internalised stigma. Thus, additional systematic research with existing ads is needed to determine the nature and extent of these potential effects (both in terms of short and long-term consequences) prior to recommendations being made about specific types of messages that may be most promising to pursue in future population-based obesity prevention mass media campaigns. This further research should test ads that focus on body weight as well as ads that emphasise specific health behaviours (e.g. healthy eating or physical activity) without mentioning weight, in order to enhance the evidence base regarding the comparative effects of these different approaches to health promotion. Finally, it is essential that newly developed obesity prevention ads undergo careful pre-testing, including assessment of possible stigmatising effects among vulnerable populations, prior to them being used in a campaign.

Potential methods for reconciling differing expert opinions on eating disorders and obesity have been proposed, and an integrated approach to preventing the spectrum of problems related to eating and weight (i.e. eating disorders, obesity, and unhealthy weight loss practices) has been recommended [25, 26]. This literature offers important insights into potential messaging opportunities in developing future public health advertising on this subject. Future research should examine whether it is possible to create obesity prevention advertisements that are perceived to be effective and believable to at least as many viewers as the ads that tested most favourably in this study, whilst minimising any potential stigmatising effects of the advertisements. Collaboration between experts on public health, obesity, body image and disordered eating could prove fruitful in this regard.

### Strengths and other limitations

A strength of the present study was the use of a standard, well-tested protocol that enabled different ads to be compared on an equal footing, with advertising exposure held constant across participants. It is possible, though, that people’s reactions to ads after forced exposure may not be a true reflection of how they would respond in a naturalistic media environment, where ads are typically viewed in a relatively cluttered media environment on repeated occasions over a longer period. This study assessed short-term cognitive and emotional responses to brief advertising exposure, rather than behavioural changes, which tend to occur in response to higher ‘doses’ of advertising exposure [35–37]. Participants were recruited from a national online panel with demographic quotas applied, thus the age profile of our sample was not representative of the population. However, as the main purpose of the study was to assess the relative effectiveness of obesity prevention ads (i.e. how they performed in comparison to each other) rather than the effectiveness of each ad individually, population representativeness was not vital. Randomisation processes were implemented to achieve comparable groups across ads, and demographic factors were controlled for in our multivariate analyses. A further limitation of the study was that owing to the small number of existing public health ads addressing obesity prevention, ads varied on some characteristics that would have ideally been held constant. Consequently, the finding that the two ads that tested best were produced in Australia could have been partly attributable to the Australian audience identifying more with these ads than the ads with non-Australian accents, even though participants were instructed to try to disregard such cultural differences in the ads. However, this line of argument is countered by the fact that some other Australian ads, that contained supportive/encouraging messages and animation or depicted scene execution styles, did not achieve such high scores as the Australian ads that focused on negative health consequences and used graphic imagery. Further research could explore whether such ads also perform well with audiences of different nationalities [13].

## Conclusions

This study represents an important first step in building an evidence base concerning the types of messages and executional styles that could be further pursued as part of effective obesity prevention mass media campaigns. Overall, the findings are congruent with the anti-smoking advertising literature in that they suggest that ads presenting information regarding the health consequences of excess weight appear most persuasive, and that the use of graphic imagery has most potential to emotionally resonate with audiences. Further research that extends beyond assessing initial, short-term responses to measuring actual behaviour changes is needed to provide stronger support for the efficacy of such ads in promoting healthy weight. In addition, it is imperative that new messages designed to emphasise the serious health consequences of excess weight are carefully pre-tested prior to being aired to ensure they do not have unintended negative impacts such as increased stigmatisation of vulnerable individuals and increased levels of body dissatisfaction and/or eating-disordered behaviour among at-risk population sub-groups.

## Additional file

Additional file 1:
**Obesity prevention advertisement descriptions.** (DOC 71 kb)
