# Supplementary material for: Finding the keys to successful adult-targeted advertisements on obesity prevention: an experimental audience testing study
Source: BMC Public Health. 2015 Aug 20;15:804. doi: 10.1186/s12889-015-2159-6 (PMC4546051; doi:10.1186/s12889-015-2159-6)
Supplement: Additional file 1: — Obesity prevention advertisement descriptions. (DOC 71 kb) [file 12889_2015_2159_MOESM1_ESM.doc]

| **Become a swapper**  *Description:* This animated ad features a blue balloon character called Eric who has gradually become overweight. He explains how he is reducing his waistline and risk of chronic disease by swapping “more for less” and “inside for outside”.  *Source:* Australian Government  <http://www.youtube.com/watch?v=eDYlw4vRMNE&feature=related> |
| --- |
| **Toxic fat**  *Description:* This ad begins with a man at home opening his fridge and pulling out a piece of pizza for a snack. As he grabs the fat around his waist, the visuals go inside his body to show fat around his organs, with a voiceover explaining that this “toxic fat’ is linked with serious chronic diseases.  *Source:* Western Australian Department of Health, National Heart Foundation (WA division) and Cancer Council Western Australia  <http://www.youtube.com/watch?v=pThTr83UWa8> |
| **Take life on**  *Description:* This ad features a woman walking along and becoming visually healthier as she talks about making simple decisions to choose healthier eating and activity options. A voiceover explains how small lifestyle changes can reduce chronic disease risk and improve self-esteem.  *Source:* Scottish Government  <http://www.youtube.com/watch?v=exJok3jqpyQ&feature=plcp> |
| **Measure up**  *Description:* This ad depicts a young man walking along an oversized tape measure, progressively ageing and gaining weight. As he struggles to keep up with his daughter, a graphic appears on screen showing how waistlines over a certain size increase chronic disease risk. A voiceover says “The more you gain, the more you have to lose”.  *Source:* Australian Government  <http://www.youtube.com/watch?v=UWTlHqca5AM> |
| **Piece of string**  *Description:* This ad depicts a domestic scene with a young girl measuring two pieces of string to the target waist circumferences specified by the voiceover. Her unsuccessful attempt to wrap the long string around her father’s waist indicates he has a waistline that increases his risk of various cancers.  *Source:* Cancer Council Victoria  <http://www.youtube.com/watch?v=upi0EKiKtq8> |
| **Full monty**  *Description:* This ad begins with a man eating leftovers off his workmate’s plate, followed by a scene of a teenage boy choosing not to eat a bag of potato crisps like his overweight father is doing. It finishes with a man in the shower looking down at his oversized stomach, followed by the tagline “Choose to see the full monty”.  *Source:* Scottish Government  <http://www.youtube.com/watch?v=E44J-K_0WGU&feature=plcp> |
| **Correctly identified**  *Description:* This ad features a red line across the screen with “healthy”, “overweight” and “obese” written across it. As different people appear one at a time, the white box slides between the three weight categories, with a voiceover encouraging viewers to go to their website to find out which category fits them.  *Source:* LiveWell Colorado  <http://www.youtube.com/watch?v=pNnPE2nheZ8&feature=relmfu> |
| **Why am I fat**  *Description:* This ad shows a mother and son, who are both overweight, sitting down opposite each other and the son asking “Mum, why am I fat?”. Text on screen states that “75% of Georgia parents with overweight kids don’t recognise the problem” followed by the tagline “Stop sugarcoating it, Georgia”.  *Source:* Children’s Healthcare of Atlanta  <http://www.youtube.com/watch?v=ysIzX_iDUKs> |
